# NURSING HOME SOCIAL WORK: CONFLICTING VALUES AND ETHICAL PRACTICE WITH RESIDENTS, FAMILIES, AND STAFF

**DOI:** 10.1093/geroni/igad104.0105

**Published:** 2023-12-21

**Authors:** Mercedes Bern-Klug, Denise Gammonley

## Abstract

People who live in nursing homes and those who spend weeks or months there recuperating from a hip replacement or stroke have a broad range of needs, many urgent and others enduring. Despite the high needs of residents—which have increased over the past decade as resident acuity has increased-- the settings are typically under-staffed both in terms of the number of direct care staff and the preparation of direct care staff (NASEM Report, 2022). Social workers and others in the social service role (i.e., those without a social work education) step into or are tapped to intervene when resident rights conflict with family wishes and/or staff rights and wishes. In this session using findings from both qualitative and quantitative studies, we will describe how and what type of conflicts social workers are involved with and the training they report they need to be better prepared to serve as a resource in this important role.

